# Characterisation of P-glycoprotein-9.1 in *Haemonchus contortus*

**DOI:** 10.1186/s13071-016-1317-8

**Published:** 2016-01-28

**Authors:** Pablo Godoy, Hua Che, Robin N. Beech, Roger K. Prichard

**Affiliations:** Institute of Parasitology, Macdonald campus, McGill University, 21,111 Lakeshore Road, Sainte Anne-de-Bellevue, H9X3V9 QC Canada

**Keywords:** Macrocyclic lactone resistance, P-glycoprotein, Transport studies, Heterologous expression system, Protein localisation, Ivermectin, Abamectin, Moxidectin

## Abstract

**Background:**

The existence nematodes of veterinary importance such as *Haemonchus contortus* resistant to anthelmintic drugs, including the macrocyclic lactones, has become a major concern in animal health. Macrocyclic lactone resistance in *H. contortus* seems to be multigenic including the active efflux of these drugs by P-glycoproteins, members of the ABC transporter family, present in this parasite. The goals of the present work were to determine the activity of *H. contortus* P-glycoprotein 9.1 (*Hco*-PGP-9.1) and its interaction with the avermectins, ivermectin, abamectin, and also the milbemycin, moxidectin. Additionally, the localisation of *Hco*-PGP-9.1 was sought in adult worms.

**Methods:**

*Hco*-*Pgp-9.1* was cloned and expressed in mammalian cells and its expression profile was determined at the transcriptional and protein level by qRT-PCR and Western-blot, respectively. The nematode transport activity was assessed using the tracer dye Rhodamine 123. A ligand competition assay between different macrocyclic lactones and Rhodamine 123 was used to establish whether or not there was interaction between *Hco*-PGP-9.1 and the avermectins (abamectin and ivermectin) or moxidectin. In addition, immunostaining was carried out to localise *Hco*-PGP-9.1 expression in the transgenic cells and in adult female parasites.

**Results:**

*Hco*-PGP-9.1 was expressed in the cell membrane of the transfected host cells and was able to extrude Rhodamine 123. Ivermectin and abamectin, but not moxidectin, had a pronounced inhibitory effect on the ability of *Hco*-PGP-9.1 to transport Rhodamine 123*.* Antibodies raised against *Hco*-PGP-9.1 epitopes localised to the uterus of adult female *H. contortus*.

**Conclusions:**

These results suggest a strong interaction of the avermectins with *Hco*-PGP-9.1. However, possibly due to its physico-chemical properties, moxidectin had markedly less effect on *Hco*-PGP-9.1. Because of the greater interaction of the avermectins than moxidectin with this transporter, it is more likely to contribute to avermectin resistance than to moxidectin resistance in *H. contortus*. Possible over expression of *Hco*-PGP-9.1 in the female reproductive system in resistant worms could reduce paralysis of the uterus by macrocyclic lactones, allowing continued egg release in drug challenged resistant worms.

**Electronic supplementary material:**

The online version of this article (doi:10.1186/s13071-016-1317-8) contains supplementary material, which is available to authorized users.

## Background

Anthelmintic resistance (AR) in gastro-intestinal (GI) nematodes such as *Haemonchus contortus*, has become a serious problem for livestock industries. In small ruminants, *H. contortus* can significantly decrease flock production [1]. The primary means of parasite control in animal health is through anthelmintic drugs, of which, the macrocyclic lactones (MLs) are widely used, with activity against both ecto- and endoparasites [2]. Ivermectin (IVM), the first ML anthelmintic commercially developed, is a chemically modified product from *Strepmomyces avermitilis* fermentation [3]. The MLs have two subclasses: one corresponding to the avermectins, such as IVM, abamectin (ABA), doramectin (DOR), eprinomectin (EPR), selamectin (SEL) [4] which are used to control nematodes and some ectoparasites in animals. IVM is also used in humans. Emamectin (EMC) is used in fish farming to control sea lice [5]. The other group corresponds to the milbemycins including moxidectin (MOX) and milbimycin oxime (MO), used against parasitic nematodes in farm and companion animals [6–8]. The MLs act by binding irreversibly to glutamate-gated chloride channels (GluCls), which are present in invertebrates such as nematodes, producing a flaccid muscular paralysis [9]. Structurally the MLs have a macrocyclic lactone ring based in a benzofuran core [10] but have some differences, notably the attached disaccharide moiety at the C-13 of the ML ring present in the avermectins but not on the milbemycins [11]. In addition, the milbemycin, MOX has a methoxime at C-23 and an olefinic side chain at C-25, which are not present in the avermectins [2]. The use of the ML in veterinary medicine has been the anchor strategy to control parasitic pathogens but also in the case of IVM, has been applied successfully to control human parasitoses such as river blindness disease caused by *Onchocerca volvulus* [12]. Despite the remarkable ML efficacy, parasitic nematodes have developed resistance to these drugs, limiting their parasiticidal effect [13, 14]. In large animal nematodes such as *H. contortus*, ML resistance has been reported in field isolates and experimentally selected strains [15–18]. ML resistance in nematodes has a genetic basis involving several genes turning into a complex process not completely understood [2]. One of the mechanisms hypothesised to be participating in ML resistance in nematodes is possibly the active transport of these drugs by the ATP-Binding-Cassette (ABC) transporters such as P-glycoprotein (PGP) [19]. ABC transporters are cell-membrane proteins that transport across the membrane, a wide range of structurally unrelated substrates, including anthelmintics such as IVM [20]. In nematodes, including *H. contortus* and the free-living model *Caenorhabditis elegans*, an up-regulation of ABC transporters that has a direct correlation with a ML resistance phenotype has been reported [21, 22]. Moreover, in *H. contortus* some P-gps have been reported to be over-expressed in IVM field resistance strains, including *Hco-Pgp-2* or A [21–23] and also *Hco-Pgp-9* or E [23]. Recently, the *H. contortus* genome has been fully sequenced, describing at least 10 *Hco-Pgp* genes among the ABC transporters present in this parasite [24]. In *H. contortus*, three copies of *Hco-Pgp-9* gene (*Hco-Pgp-9.1*, *Hco-Pgp-9.2* and *Hco-Pgp-9.3*) have now been partially identified [24], with expression of the *Hco-Pgp-9.1* transcript, previously termed *Hco-Pgp-*E [23], being reported to be up-regulated in ML selected and field resistant strains of *H. contortus* [23]. Regarding orthologs in other trichostrongylids from ruminants, an up-regulation of *Tci-pgp-9* in all life-cycle stages of the sheep nematode *Teladorstagia circumcinta* in IVM resistant isolates has been described [25]. In another helminth from cattle, *Cooperia onchophora* an up-regulation of the ortholog *Con-*pgp*-9* in an IVM selected strain was also described [26].

Due to this data showing an over-expression of *Hco-Pgp-9.*1, and its orthologs, in *H. contortus* and other nematodes with veterinary importance resistant to ML, as well as the strong interaction described between *Hco*-PGP-2 and the avermectins, suggesting a nematode transporter involvement in ML resistance in *H.* contortus [27], the aim of the present work was to characterise the *Hco*-PGP-9.1 transport activity and its interaction with the MLs, in order to ascertain its possible involvement in the resistance to these drugs. In addition, *Hco*-PGP-9.1 expression and localisation in the adult stage of this parasite was investigated.

## Methods

### Ethics statement

Animals and standard operating procedures (SOPs) used in this research study were approved (Protocol #3845) and adhered to the guidelines from the Animal Care Committee of McGill University.

### Worms

Adult *H. contortus* from the laboratory PF23 isolate, susceptible to the MLs [15], were used. Worms were collected from the abomasum of sheep and incubated in PBS (phosphate-buffer saline) for 2 h at 37 °C.

### Cells and reagents

An aliquot of adherent parental LLC-PK1 cells (pig kidney epithelium) and a transgenic cell line over-expressing the *mdr1a* gene (mouse PGP) called *mdr1a*/LLC-PK1 as positive control for transport studies, were gifts from Dr. A. H. Schinckel (The Netherlands Cancer Institute, The Netherlands). HBSS media, 199 media, Lipofectamine 2000®, G418, penicillin/streptomycin, TOP10F’competent cells, Trizol®, pcDNA 3.1(+) mammalian expression vector, Rhodamine 123; secondary antibodies Alexa fluor 488®, Alexa fluor 633 F(ab’)_2_, DAPI (4′,6-diamidino-2-phenyldole) staining and the BCA protein assay kit were purchased from Thermo Fischer Scientific (Burlington, ON, Canada). RNeasy® kit, Omniscript® reverse-transcription kit, and SYBR Green were obtained from Qiagen (Hilden, Germany). Subcloning vector pGMET-Easy was from Promega (Madison, WI, USA). Restriction enzymes *BamH I* and *Not* I were from New England Biolabs (Ipswich, MA, USA). MOX was a kind gift from Wyeth (Fort Dodge Animal Health, Madison, NJ, USA). IVM and ABA and all the listed chemicals were from Sigma-Aldrich (Burlington, ON, Canada). SDS-PAGE and Western-blot reagents were from BIO-RAD (Hercules, CA, USA). The chemiluminescence kit was from GE Life Sciences (Mississauga, ON, Canada).

### RNA extraction

Worms were homogenised and lysed with the Trizol® reagent according to the manufacturer’s instruction. The RNA pellet obtained was dried and eluted in 50 μl of RNase free water. RNA quantification was measured in a nano-photometer IMPLEN® (Westlake Village, CA, USA) at 260 nm wavelength. All extracted RNA was kept at -80 °C.

### Reverse transcription polymerase chain reaction (RT-PCR)

Total RNA (1 μg) was reverse transcribed using the Omniscript® reverse transcription kit following the manufacturer’s protocol. Synthesised cDNA was stored at–20 °C for further use.

### Amplification and cloning of the full length *H. contortus Hco-Pgp-9.1* cDNA sequence

*H. contortus* P-glycoprotein-9.1 DNA sequence was from ([28], Additional file 1). Specific primers to amplify the full-length sequence were designed using Geneious bioinformatics software, version 5.5.6 (Biomatters Ltd, Auckland, New Zealand). The cloning strategy was to ligate the *Hco-Pgp-9.1* cDNA sequence and the linearised pcDNA3.1 (+) mammalian expression vector using restriction sites. To carry this out, the primers used were: forward primer 5′-aagttagggatccc**C**acc**ATGG**GCTTTTTGAAGAACGG-3′ including a specific sequence for pcDNA3.1 (+) in small case, then a *BamH I* restriction site (underlined in small case), after a Kozak sequence, around the start codon, both in bold [29]*.*

Reverse primer 5′-agaccta**gcggccgc**TCATCCCTTGGCCAATGTTTGCTT-3′, included a *Not* I restriction site (in bold small case), then a stop codon (underlined as above). A PCR with these primers was run. After confirmation that the expected amplicon was present, the PCR product was ligated into pGEM-T**®** Easy subcloning vector. Furthermore, the cloned sequence was digested individually with each restriction enzyme referred above, and ligated into pcDNA 3.1(+) mammalian expression vector using the same restriction sites.

Transformation was carried out in *E. coli* TOP10F’ competent cells. After, positive colonies were screened by colony PCR and restriction digestion analysis. DNA extracted from positive transformants was digested with *BamHI* and *Not* I, confirming the construct ligation and these DNA plasmids were sent for sequencing at Genome Quebec Innovation Centre (McGill University, QC, Canada) to confirm the specific *Hco-Pgp-9.1* full-length cDNA sequence.

### Stable transfection in mammalian cells

For stable transfection the mammalian porcine cell line LLC-PK1 was used [27]. Original LLC-PK1 parental cells were seeded on 24 well plates (2 × 10^5^ cells/well) and grown in 199 medium supplemented with 10 % fetal calf serum and penicillin/streptomycin (100 units/ml; 100 μg/ml, respectively). Cells close to 100 % confluency were transfected using 18 μg of plasmid DNA using Lipofectamine 2000®, following the manufacturer’s instructions. In parallel, as a positive control for transfection, the Chloramphenicol Acetyl Transferase (CAT) gene was transfected into these cells. Twenty-four hours after transfection, medium was removed and replaced with supplemented 199 medium. Forty-eight hours after transfection, the cells were washed, collected and counted in order to seed them to 25 % confluency in 24 well plates under selection pressure of G418 or neomycin (Geneticin®) at 400 mg/l.

After several weeks of selection, stable transfected colonies were collected individually and transferred to new culture flasks. Each individual colony was assessed for growth rate, morphological shape and survival under selection with G418.

### Expression profile characterisation

#### Transcript profile: qRT-PCR

RNA was extracted from stable transfectants using the RNeasy extraction kit, following the manufacturer’s instructions as above. Subsequently, a reverse transcription-PCR was done with the extracted RNA. Then, the synthesised cDNA was used as template for a qRT-PCR with specific primers (see Table 1) for *Hco-Pgp-9.1* and the pig *mdr1* (GenBank accession number XM_003130205.3). Pig GAPDH (Glyceraldehyde-3 phosphate dehydrogenase) was used as a housekeeping gene for normalisation (GenBank accession number NM_001206359.1). After serial dilution of the cDNA template, a qRT-PCR was carried out using SYBER Green in a Rotor-Gene thermal cycler (Qiagen, Hilder, Germany) with the following conditions: initial hold at 95 °C for 15 min, then 40 cycles including: 94 °C for 15 s; 57 °C for 30 s for annealing and 72 °C for 1 min final extension. Subsequently, a melting curve analysis was done for all the amplified PCR products. Primers for target and housekeeping genes were run in four replicates. After every qRT-PCR run, data were analysed and the efficiency of the reaction estimated according to [30]. Relative expression of *Hco-Pgp-9.1* over the endogenous pig *mdr1* was obtained based on the 2^ΔΔ^ Ct method.

**Table 1:** Primers for qRT-PCR (quantitative Real-Time-PCR) on *Haemonchus contortus* P-glycoprotein-9.1/epithelial-like pig (*Sus scorfa*) kidney cell (*Hco*-*Pgp-9.1*/LLC-PK1) transfected cells

Primer name	Nucleotide sequence (5′–3′)
*Hco-Pgp-9.1* (forward)	GGC CTC AGT TTG CTG TTC TC
*Hco-Pgp-9.1* (reverse)	ATC TGG TCG CGT TGG ATA AG
*Sus scorfa GAPDH* (forward)	AAC TGC TTG GCA CCC CTG
*Sus scorfa GAPDH* (reverse)	TTG GCA GCG CCG GTA GAA
*Sus scorfa mdr1* (forward)	TGC CAC CAC GAT AGC TGA GAA CAT
*Sus scorfa mdr1* (reverse)	ATG GCG ATT CTC TGC TTC GTC CA

#### Protein profile: membrane enrichment fraction

*Hco-Pgp-9.1*/LLC-PK1 transfected cells were grown in 75 cm^2^ flasks close to 100 % confluency. Cells were collected with a scraper, counted and re-suspended in buffer containing protease inhibitors (1X PBS pH 7.4; 1 mM PMSF; 1 mM EGTA; 10 μg/ml Leupeptin; 1 μg/ml Pepstatin A). Cells were then washed twice with a solution containing 1X PBS + 1 mM DTT + 1 mM PMSF to a volume of 50 ml. Then the cell suspension was divided into two tubes (25 ml volume each) and centrifuged at 1000× g for 15 min at 4 °C. Afterwards, each pellet was re-suspended in an appropriate volume of buffer containing 1X PBS + 1 mM DTT + 1 mM PMSF. Cells were then sonicated 3 times for 10 s using 40 % amplitude. Subsequently, cell pellets were centrifuged at 1400× g for 15 min at 4 °C. Supernatants were carefully collected and combined in a single tube. This sample was ultra-centrifuged at 135 000× g for 60 min at 4 °C. The supernatant was discarded and the pellet re-suspended in 2.5 ml of a solution containing 1X PBS + 1 mM DTT + 1 mM MgCl_2_. Finally, the protein concentration from isolated membranes was quantified by the BCA protein assay method and kept at–80 °C.

In parallel, an aliquot of whole crude membrane from *H. contortus* worms was isolated. Fifty *H. contortus* adult worms (females and males) were homogenised in buffer containing PBS 1× pH 7.4; 1 mM PMSF; 1 mM EGTA; 10 μg/ml Leupeptin and 1 μg/ml Pepstatin A. The solution was centrifuged at 45 000× g for 15 min at 4 °C, the supernatant was recovered and centrifuged again at 45 000× g for 15 min at 4 °C. The supernatant was carefully recovered and sonicated 3 times for 10 s with 40 % amplitude. Finally, the sample was ultracentrifuged at 135 000× g for 60 min at 4 °C, the pellet was recovered and re-suspended in PBS + 1 mM DTT + 1 mM MgCl_2_ + 0.5 % Triton X-100 for further protein quantitation by the BCA protein assay method. Samples were kept at–80 °C until further separation on SDS-PAGE.

### *Hco*-PGP-9.1 antibody preparation and Western blot

Peptide antigens were selected from the *H. contortus* PGP-9.1 amino acid sequence, analysed, synthesised commercially and used to immunise rabbits by 21^st^ Century Biotech. Inc. (Marlborough, MA, USA) (see Additional file 1). The specificity of purified *Hco*-PGP-9.1 peptide epitopes was determined by ELISA and dot blot. A mixture 1:1 of both antibodies for each antigen peptides for *Hco*-PGP-9.1 was used for further Western-blot and immunolocalisation. Membrane protein fractions previously obtained from *Hco-Pgp-9.1*/LLC-PK1, CAT/LLCPK1 control cells and *H. contortus* worms whole crude membrane extracts, from a mixed population of female and male adult worms, were separated in a 4.5–10 % polyacrylamide gradient gel. Proteins were transferred, by the semi-dry system, to a PVDF membrane at 25 volts for 25 min. The membrane was blocked overnight at 4 °C in 5 % skim milk blocking solution with 0.05 % Tween-20®. Then the membrane was washed thoroughly in TBS-0.05 % Tween-20®. The membrane was then incubated, overnight at 4 °C, with anti-*Hco*-PGP-9.1 antibody, at 1/500 dilution.

After several washes, the membrane was incubated with anti-rabbit secondary antibody conjugated to horseradish peroxidase in a 1/5000 dilution at room temperature for 1 h. After washing, the membrane was developed using a chemiluminescence kit and X-Omat films.

### Immunofluorescence assay (IFA) on *Hco-Pgp-9.1*/LLC-PK1 transfectants

*Hco-Pgp-9.1*/LLC-PK1 transfectants were grown on coverslips to 70 % confluency. Cells were then fixed with 4 % paraformaldehyde (PAFOH). The cells were washed and blocked with 3 % BSA for 1 h at room temperature, and then washed and incubated with the anti-*Hco*-PGP-9.1 antibody, or anti-*Hco*-PGP-9.1 antibody pre-absorbed by both synthetic peptides (pre-absorbed negative control), at 1/50 dilution overnight at 4 °C in the dark. The cells were then incubated with Alexa®Fluor 488® secondary antibody in 1/3000 dilution for 45 min at room temperature in the dark. Cells were then washed and incubated with nucleic DAPI medium for 4 min at room temperature in the dark. Following this, the cells were washed and mounted on a slide with mounting media and sealed with nail polish. After overnight incubation, stained cells were examined under an ECLIPSE T*i* inverted epifluorescence microscope (Nikon, Melville, NY, USA) at λ488nm excitation and λ519nm emission. Images obtained with different dyes were merged using ImageJ software (http://imagej.nih.gov/ij/).

### Transport assays in transgenic cells expressing *Hco-Pgp*-9.1 with Rhodamine 123 and macrocyclic lactones

In order to measure the nematode transporter activity, *Hco-Pgp-9.1*/LLC-PK1 transfected cells were tested with Rhodamine 123 (Rho123) [11] as substrate for efflux translocation. Cells were plated in 24 well plates in G418-free medium until confluent. Then, cells were incubated in HBSS media, 1 % BSA containing 10 μM Rho123 with or without increasing concentrations of the endectocides: IVM, ABA or MOX (0.00625–20 μM). Additionally, the cyclosporin A analogue, valspodar (VSP or PSC833) (kindly provided by Novartis Animal Health, Switzerland) was used, at 5 μM, as a maximum reference inhibitor of *Hco*-PGP-9.1 transport function since this compound is a slow transport substrate for MDR1.

All the drugs were dissolved in DMSO and diluted in HBSS transport media with a final DMSO concentration of < 0.2 %. *Hco-Pgp-9.1*/LLC-PK1 transfected cells were incubated with all the reagents, including Rho123 for 2 h. After incubation, the medium was discarded and cells carefully washed three times with PBS. Finally cells were lysed in PBS/0.5 % SDS. The lysates were stored at–20 °C until further analysis.

### Fluorescence measurement

The intracellular accumulation of Rho123 was measured using a FLUOstar Galaxy fluorimeter (BMG LABTECH, Ortenberg, Germany). The reading parameters for Rho123 were λmax = 507 nm excitation and λmax = 529 nm emission. The inhibition of the *Hco*-PGP-9.1 transport function by the MLs was compared against the maximum effect produced by valspodar [11] and expressed as percent of total VSP inhibition. Each experiment was repeated using three biological replicates and each drug concentration was run in triplicate. The data generated were fitted using GraphPad Prism software (Version 6).

### Localisation of *H. contortus* P-glycoprotein-9.1 in adult worms

The *Hco*-PGP-9.1 antibody mixture described above was used for immunolocalisation of *Hco*-PGP-9.1 expression in the *H. contortus* adult stage. Worms were collected and fixed in 3.7 % (w/v) paraformaldehyde in PBS at 4 °C for 6–12 h. Worms were then washed three times in PBS for 15 min and stored at 4 °C in 0.1 % (w/v) paraformaldehyde in PBS. Subsequently, worms were incubated overnight at 37 °C in 5 % (v/v) 2-mercaptoethanol, 1 % Triton X-100, 125 mM Tris–HCl pH 6.9, and washed briefly in PBS. Fixed worms were digested for 6–20 h until complete removal of the cuticle layer, in 120 U/ml collagenase at 37 °C in 1 mM CaCl_2_, 100 mM Tris–HCl pH 7 [31]. Worms were then washed in PBS and incubated for 72 h at 4 °C with a 1/200–300 dilution of the primary antibody in PBS containing 0.1 % (w/v) BSA, 0.5 % (v/v) Triton X-100 and 0.05 % (w/v) sodium azide. Non-specific binding was removed after several washes in PBS/0.1 % (v/v) Triton X-100 at 4 °C [32]. Worms were incubated with the secondary antibody Alexa® Fluor 633 F(ab’)_2_ far-red fluorescein goat anti-rabbit in a 1/3000 dilution at 4 °C for 15–18 h. Worms were washed in PBS containing 0.1 % (v/v) Triton X-100. Finally, worms were mounted on slides using mounting medium and examined under the confocal microscope (laser Bio-Rad Radiance®2100 MP) at λ632 nm emission and λ647 nm excitation.

## Results

### Expression profile of *Hco-Pgp-9.1* in transfected cells

**Fig. 1:**
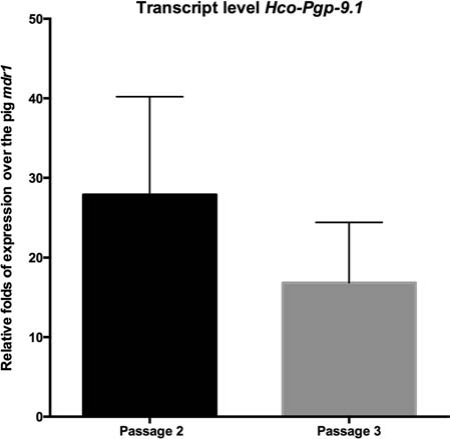
qRT-PCR on *Hco-Pgp-9.1*/LLC-PK1 cells. *Hco-Pgp-9.1* transcript level present in the transfected pig host cells corresponding to cell culture passages 2 and 3. Relative expression over pig *mdr1* endogenous gene, calculated by the 2^ΔΔ^ Ct method using the pig GADPH transcript as reference. Mean of 4 technical replicate experiments for each passage ± SD

**Fig. 2:**
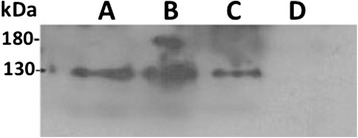
Western blot using anti-*Hco*-PGP-9.1 antibody. Lane (**a**) *Hco-Pgp-9.1* membrane extracted from *Hco-Pgp-9.1*/LLC-PK1 transfected cells, clone 6. Lane (**b**) whole crude membranes extracted from *Haemonchus contortus* adult worms. Lane (**c**) *Hco-Pgp-9.1* membrane extracted from *Hco-Pgp-9.1*/LLC-PK1 transfected cells, clone 9. Lane (**d**) CAT (Chloramphenicol acetyl transferase)/LLC-PK1 membranes (control cells). Each lane was loaded with 5 μg of protein

The relative expression of *Hco-Pgp-9.1* in mammalian LLC-PK1 cells is shown in Fig. 1. The *Hco-Pgp-9.1* transcript was expressed at 30 fold the level of endogenous pig *mdr1* present naturally in this porcine origin cell line at the second passage of these transfectants. The expression of the *Hco-Pgp-9.1* transcript appeared to be decreasing by the third passage of the same culture (although the differences were not statistically significant), but the difference compared with the endogenous pig *mdr1* was still large. The expression profile was also corroborated at the protein profile by Western blot. A protein band of 130 kDa molecular weight was observed from membranes extracted from *Hco-Pgp-9.1*/LLC-PK1 transfectant clones (Fig. 2, lanes A and C). In the whole crude membranes extracted from *H. contortus* adult worms (lane B), a similar 130 kDa band and a higher molecular size band close to 180 kDa were seen. The protein band of 130 kDa identified in transfected cells may correspond to the unglycosylated form of *Hco*-PGP-9.1, whereas the whole crude membranes from the adult stage of the parasite, may contain both unglycolysalted (130 kDa) and glycosylated (~180 kDa) forms of *Hco*-PGP-9.1.

**Fig. 3:**
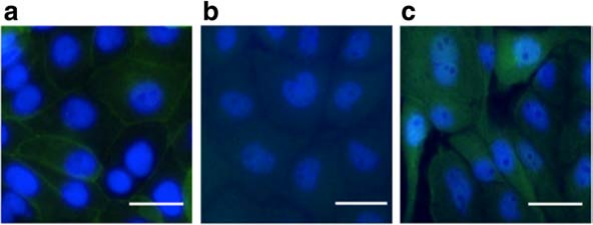
Immunofluorescence assay (IFA) on *Hco*-Pgp-9.1 transfected cells (**a**). Anti-*Hco*-PGP-9.1 antibody conjugated with fluorescein (AlexaFluor® 488) recognised a strong signal of *Hco*-PGP-9.1 in the plasma membrane of the host cells (green). **b** *Hco-Pgp-9.1*/LLC-PK1 cells incubated with peptide pre-adsorbed *Hco*-PGP-9.1 antibody as control. **c** CAT/LLC-PK1 control cells incubated with anti-*Hco*-PGP-9.1 antibody. Epifluorescence microscopy image, magnification: 40X. DAPI stain (blue) of cell nuclei. *Scale-bar*, 50 μm

The expression of this nematode transporter in the host cells was also supported by the subcellular localisation of *Hco*-PGP-9.1 in the plasma membrane of the transfected cells (Fig. 3a). *Hco-pgp-9.1* transfected cells exposed to anti-*Hco*-PGP-9.1 antibody that had been pre-absorbed with the epitope peptides (Fig. 3b) did not show staining in the cell membrane. The CAT/LLC-PK1 control cells (Fig. 3c) exposed to the anti-*Hco*-PGP-9.1 antibody (not pre-absorbed) showed generalised fluorescence in the cytoplasm, possibly consistent with autofluorescence or an interaction with the CAT enzyme expressed in the control cells. However, these control cells did not show labelling of the plasma membrane as in the *Hco-Pgp-9.1* transfected cells treated with the same antibody. A similar generalised fluorescence of the cytoplasm of CAT transfected cells was previously observed (27), using the same secondary antibody, but a different primary antibody, suggesting that this generalised fluorescence of the CAT transfected cells is non-specific.

### Transport studies of *Hco*-PGP-9.1

**Fig. 4:**
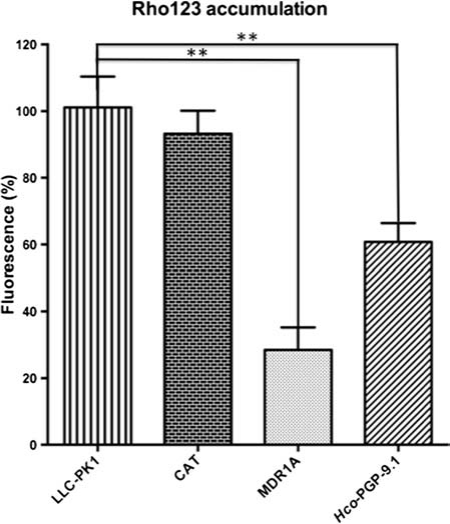
Functional transport assay using the fluorophore probe Rhodamine 123 (Rho123). Fluorescence in control cells (untransfected LLC-PK1, and CAT transfectants); cells over expressing the mouse PGP (MDR1A– positive control) and the nematode transporter cells (*Hco-*PGP-9.1). Results are means of 3 biological replicate experiments ± SD. Analysis by unpaired, two-tailed *t*-test showed both the MDR1A and PGP-9.1 were significantly different from the untransfected LLC-PK1 cells, *P* < 0.0001 (**)

**Fig. 5:**
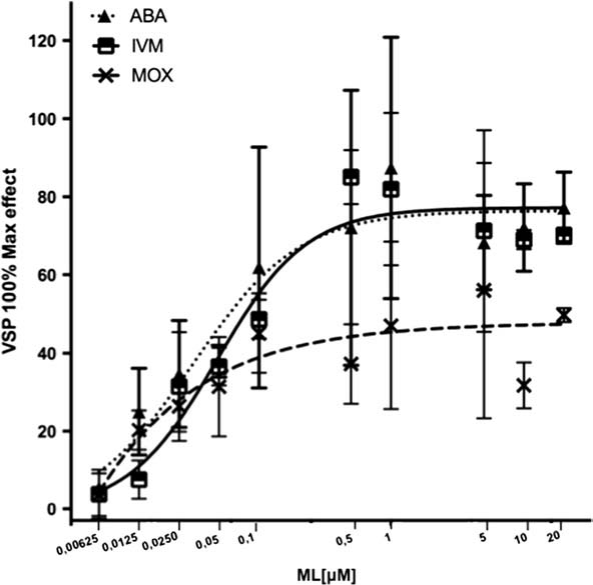
Inhibition by MLs of Rhodamine123 transport in *Hco*-PGP-9.1 transfected cells. Effect of abamectin (ABA), ivermectin (IVM) and moxidectin (MOX) compared with the maximum effect displayed by valspodar (VSP), 5 μM. Results are means of three independent biological replicate experiments ± SD

After the *Hco*-PGP-9.1 expression profile was assessed, *Hco-Pgp-9.1/LLCPK1* transfected cells were subjected to a Rho123 translocation assay. It was found that *Hco-Pgp-9.1/LLCPK1* transgenic cells accumulated less than 40 % Rho123 fluorescence compared with the control parental cells (LLC-PK1) and CAT/LLC-PK1 cells (Fig. 4), indicative of Rho123 efflux by the transfectants expressing *Hco*-PGP-9.1. Although Rho123 fluorescence accumulation was higher in *Hco-Pgp-9.1/LLCPK1* transfectants than in positive control cells over expressing *mdr1a* (mouse PGP), these fluorescence means are significantly lower than in the control LLC-PK1 untransfected cells. Subsequently, a competition assay between the different MLs and the Rho123 transport by *Hco*-PGP-9.1 was run. A pronounced inhibitory effect by the avermectins (IVM and ABA) on *Hco*-PGP-9.1 Rho123 transport was found, giving a saturable effect on the inhibition of transport of this fluorophore, which was close to 80 % of the total Rho123 inhibition effect produced by VSP on *Hco*-PGP-9.1 (Fig. 5). In contrast, MOX caused a non-saturable inhibitory effect on the Rho123 transport by *Hco*-PGP-9.1. This MOX effect contrasts with the avermectin effects, for both IVM and ABA in which 0.5 μM produced a maximal inhibition of Rho123 transport corresponding to 80 % of the effect produced by VSP at 5 μM. In contrast, MOX even at 10 or 20 μM concentrations, reached only 50 % or less of the maximal VSP effect (5 μM) on the inhibition of Rho123 transport.

### Localisation of *Hco*-PGP-9.1 in *H. contortus* adult stage

**Fig. 6:**
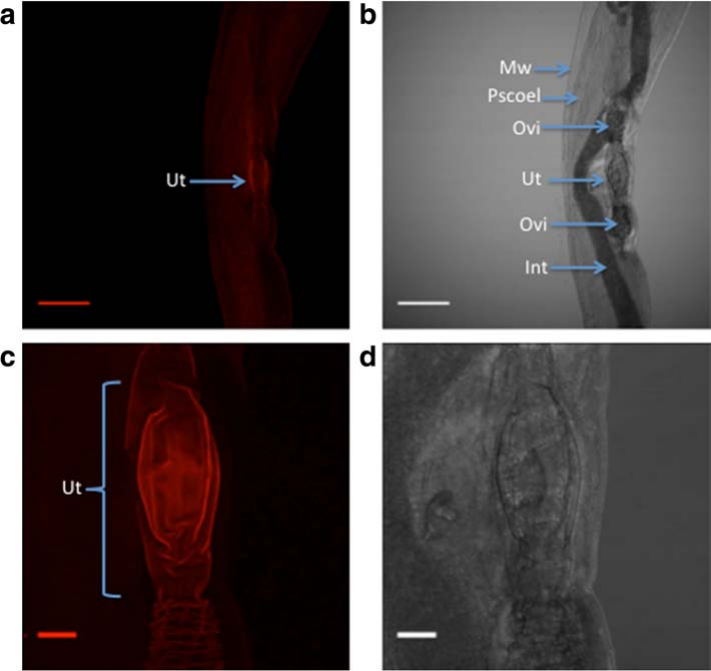
Immunolocalisation of *Hco*-PGP-9.1 in an *H. contortus* adult female worm. **a** magnification 20×, Ut: uterus. **b** magnification 20×, Mw: muscle wall, Pscoel: pseudocoelom, Ovi: oviduct, Ut: uterus, Int: intestine. **c** magnification 40×, Ut: uterus. **d** magnification 40× phase contrast for image (**c**). Scale-bars: (**a**, **b**), 50 μm; (**c**, **d**), 5 μm

The same specific antibody was used to try to determine the localisation of *Hco*-PGP-9.1 in *H. contortus* adult worms. After examining the whole body, a defined signal corresponding to the reproductive system, particularly at the level of the uterus, can be found in female worms (Fig. 6a–d).

## Discussion

ML endectocides represent the cornerstone of current chemotherapy to control different pathogens such as ticks, diptera and nematodes [33]. Resistance to these compounds has arisen particularly in trichostrongylids such as *H. contortus*, initially against IVM [34] and later against ABA and MOX [35, 36]. The involvement of ABC transporters in ML resistance in nematodes has been investigated and suggests a linkage between nematode transporters and possible efflux modulation of the MLs [37–39].

We have investigated the transport activity of *Hco*-PGP-9.1 expressed in mammalian cells and its interaction with the different MLs. An expression profile of this transporter was obtained in the transfected cells, based in its transcript and protein levels in addition to its localisation in the plasma membrane of the host cells. The expressed *Hco*-PGP-9.1 was probably unglycosylated in the host cells, based on a comparison of the molecular size of the band seen in Western blots and the predicted molecular size of the protein. This result is consistent with the *Hco*-PGP-2 expression pattern found in the same mammalian cell line and in whole crude membranes extracted from *H. contortus* [27]. Glycosylation is not required for functional activity of PGPs expressed in cells [40]. Both the unglycosylated *Hco*-PGP-9.1, and the previously characterised unglycosylated *Hco*-PGP-2 [27] were able to translocate Rho123.

The protein expression pattern in the porcine cells was similar to the one previously obtained for *Hco*-PGP-2 [27] except that the transcript level of the *Hco-pgp-9.1* transgene appeared to decrease by the third passage (the transcription was not very stable). This is probably because the *Hco-Pgp-9.1* gene, for transfection into the LLC-PK1 cells, was not codon optimised for the host pig cells as was *Hco-Pgp-2* [27].

When the *Hco*-PGP-9.1 transport function was assessed, using the fluorophore probe Rho123, a pronounced reduction of this tracer dye in *Hco-Pgp-9.1/LLC-PK1* transfectants was determined, indicative of an active transport by *Hco*-PGP-9.1. Previous work had pointed out the good substrate characteristics of Rho123 to test nematode transporter activity [28–41]. As shown in Fig. 5, the avermectins (ABA and IVM) produced a marked inhibitory effect on the Rho123 efflux by the *Hco*-PGP-9.1 transporter. In mammalian models the interaction of the avermectins with ABC transporters, has been described with ABA [42] and IVM inhibiting PGP activity [43] and MRP (Multi-drug Resistant Protein) activity [44]. This is comparable with the corresponding effect that the avermectins produced on Rho123 transport inhibition by *Hco*-PGP-2 [27] and by mouse PGP [11], confirming a strong interaction of the avermectins for both nematode and mammalian ABC transporters. On the other hand, MOX displayed a lower inhibitory effect on the Rho123 transport by *Hco*-PGP-9.1, implying a lesser interaction with this transporter. These observations are consistent with previous work describing the interaction of these compounds with *Hco*-PGP-2 [27], where the avermectins produced a saturation of the Rho123 transport activity compared with the inferior effect of MOX on Rho123 transport. These differences in the interaction with *Hco*-PGP-9.1, between the avermectins and MOX, are likely to be due to structural differences between the ML subgroups. Avermectins, in contrast to MOX, have a di- (or mono-) saccharide moiety attached to the C-13 of the macrocyclic ring, whereas MOX is protonated at this position [2, 45]. This olaeadrose moiety, in IVM and ABA, is thought to enhance the binding of the avermectins to the transporter [11–27]. On the other hand, MOX, in addition to the absence of sugar groups attached to the macrocyclic ring, has other structural features, notably the methoxime group at the level of the C-23 of the macrocyclic ring, which may reduce its interaction with nematode transporters. Another aspect which may contribute to this distinction in the interaction of the MLs with *Hco*-PGP-9.1, is a physicochemical characteristic that differs between the avermectins and MOX. In general, MLs are lipophilic drugs that need to cross membranes to reach their targets inside the parasite [20]. In this respect, MOX is more lipophilic and has a higher octanol/water coefficient (*logp*) than the avermectins [2], perhaps being retained by the lipid bilayer of the cell membrane. MOX has a longer half-life than the avermectins inside the host due to its longer residency in fat tissue [46, 47]. Recently, a nematode PGP from the equine helminth *Cylicocylus elongates*, termed *Ceg*-Pgp-9 (ortholog of *Ce-Pgp-9*), was expressed in yeast and its interaction, in a competition assay between the antifungical ketoconazole and MLs, characterised. A strong interaction with the avermectins (IVM and EPR), and a lesser interaction with MOX, was also found [48].

Our second objective was to try to localise the expression of *Hco*-PGP-9.1 in *H. contortus* adult worms. In the free-living nematode *C. elegans*, its paralogue *Cel-Pgp-9*, is predicted to be expressed in the pharynx and intestine [49]. However in *H. contortus*, there may be three paralogs of the *Cel-Pgp-9*, namely *Hco-Pgp-9.1*, *Hco-Pgp-9.2* and possibly *Hco-Pgp-9.3* [24–28]. It was reported that a differential transcript profile exists between *Hco-Pgp-9.2* and *Hco-Pgp-9.3* on the one hand, which were reported to be expressed more abundantly in the L3 stage, and *Hco-Pgp-9.1*, which was more abundant in the adult worm [28]. The primary antibodies were raised to epitopes that may be shared, at least between *Hco*-PGP-9.1 and *Hco*-PGP-9.2 (see Additional file 1). As a full sequence is unavailable for putative *Hco*-PGP-9.3, we are unable to assess whether the antibodies may also detect this putative P-glycoprotein. The anti-PGP-9.1 antibody labelled the uterus of the female reproductive system. If there are three paralogues of *Pgp-9*, each may have a different function and possible expression profile in the parasite. Adult female worms possess a developed reproductive tract, including the uterus, which may constitutively express this nematode transporter. The actual function of *Hco*-PGP-9.1, possibly in the uterus, is unknown. From the literature it is well established that in higher eukaryotes such as mammals, ABC transporters such as PGP are highly expressed in the apical epithelium membranes including the blood–brain-barrier [50, 51] and placenta [52, 53], modulating xenobiotic passage to the central nervous system or from mother to fetus. It was found that *mdr1a* (-/-) (ABCB1 knock-out) adult mice had significantly greater susceptibility to IVM neurotoxicity than wild-type mice [50]. In the CF-1 mouse strain carrying a spontaneous mutation on the *mdr1a* gene, due to the absence of PGP expressed in the placenta barrier, IVM induces a congenital disorder similar to cleft palate in mouse pups [54]. In addition, at the level of the placenta, the active transport of lipophilic molecules such as progesterone, an important female hormone during pregnancy, has been described [55]. Our finding of possible *Hco*-PGP-9.1 expression in the uterus suggests analogous functions to ABCB1 in mammals, including transport of xenobiotic products from uterine tissue, or recruitment of some lipid molecules to complete ova maturation before release from the female worm through the vulva.

In the context of ML resistance in parasitic nematodes such as *H. contortus*, the apparent localisation of *Hco*-PGP-9.1 could represent an interesting discovery. It is well known in the human filarial nematode *O. volvulus*, that IVM produces a temporary arrest of microfilaria release from the female worms [56]. In another filarial species, the heartworm of dogs, *Dirofilaria immitis*, and in the pig nematode *Oesophagostomum dentatum*, IVM interferes with embryogenesis in the adult female worms [57, 58]. Recently, a high expression of GluCl receptors, such as the *avr-14* gene, has been reported in the reproductive system of female and male worms of *Brugia malayi* [59]. The localisation of this GluCl may explain the IVM effects on reproductive tissues. A similar effect of MLs on the reproductive system of trichostrongylid nematodes has not been described. However, our data could suggest a role in which *Hco*-PGP-9.1, apparently expressed in the uterus of *H. contortus*, may be transporting these compounds, thereby protecting this tissue from the paralysing effect of avermectins on the uterine muscles and the release of nematode eggs from the female worm.

Complementary to the possible role of *Hco*-PGP-9.1 in ML resistance in *H. contortus*, there is evidence that paralogues of *Hco-Pgp-9.1*, expressed in other veterinary nematodes, may also be linked to avermectin resistance [25, 26]. The differences in the interaction of *Hco*-PGP-9.1 between the avermectins and MOX, may also explain the differences observed in the development of resistance to these drugs. ABA, IVM and MOX are among the most common MLs used in livestock to control gastro-intestinal helminths [60]. Different studies on small ruminant farms, have described a reduction in efficacy (<95 %, at the recommended dose rate), mainly to the avermectins whereas MOX was still effective or showed a higher efficacy against trichostrongylids, including *H. contortus* [61–63]. Nonetheless resistance to MOX can arise in gastro-intestinal parasites following MOX selection pressure [64, 65]. A possible contribution to the earlier and more common appearance of resistance to the avermectins could be the active efflux of these compounds by nematode transporters, such as *Hco*-PGP-9.1, compared with MOX. This could explain the differences seen in the resistance in the field between the avermectins and MOX.

## Conclusions

The present work has characterised the transport function of *Hco*-PGP-9.1, determining its pronounced interaction with the avermectins compared with MOX. A similar pattern of interaction with the MLs was found in the study of *Hco*-PGP-2 [27], indicating a possible involvement of nematode transporters in the efflux and elimination of these drugs from key tissues such as the uterus and digestive tract, thereby contributing to ML resistance in *H. contortus*.

## Additional file

Additional file 1:
**Multiple sequence alignment of** ***Hco*** **-PGP-9.1** **(GenBank accession number JX430397),** ***Hco*** **-PGP-9.2 (GenBank accession number: JX430398) and** ***Hco*** **-PGP-9.3 (GenBank accession number: JX430395).** (DOC 37 kb)

## Abbreviations

ABA: Abamectin
AR: Anthelmintic resistance
ATP: Adenosine-5′-triphosphate
BBB: Blood brain barrier
BCA: Bicinchoninic acid assay
BSA: Bovine serum albumin
BZ: Benzimidazole
°C: Degree Celsius
CAT: Chloramphenicol acetyl transferase
DAPI: 4′,6-diamidino-2-phenylindole
*Cel-Pgp-9.*: *Caenorhabditis elegans P-glycoprotein-9* gene
*Cel*-PGP-9.: *Caenorhabditis elegans* P-glycoprotein-9 protein
cDNA: Complementary deoxyribonucleic acid
C13: Carbon 13 macrocyclic lactone ring
DNA: Deoxyribonucleic acid
DMSO: Dimethyl sulfoxide
DTT: Dithiothreitol
EGTA: Ethylene glycol tetraacetic acid
ELISA: Enzyme-linked immunosorbent assay
F (ab’)_2_: Fragment antigen-binding 2
FBS: Fetal bovine serum
GAPDH: Glyceraldehyde 3-phosphate dehydrogenase
GluCl: Glutamate-gated chloride channel
GI: Gastro-intestinal
G418®: Neomycin
h: Hour
HBSS: Hank’s balanced salt solution
HC: Hua Che
*Hco-Pgp-2*: *Haemonchus contortus P-glycoprotein-2* gene
*Hco*-PGP-2: *Haemonchus contortus* P-glycoprotein-2 protein
*Hco-Pgp-9.1*: *Haemonchus contortus P-glycoprotein-9.1* gene
*Hco-Pgp-9.2*: *Haemonchus contortus P-glycoprotein-9.2* gene
*Hco-Pgp-9.3*: *Haemonchus contortus P-glycoprotein-9.3* gene
*Hco*-PGP-9.1: *Haemonchus contortus* P-glycoprotein-9.1 protein
IC_50_: Half maximal inhibitory concentration
IFA: Immuno fluorescence assay
kDa: kilo Dalton
λ: Wave length
*logp*: Octanol/water partition coefficient
IVM: Ivermectin
L3: Larval stage 3
min: Minute (s)
*mdr1a*: Murine *multidrug-resistance* gene isoform 1a
MDR1A: Murine P-glycoprotein isoform 1A
MDR: Multidrug-resistance
*mdr1*: Human multidrug *resistance gene 1*
MgCl: Magnesium chloride
ML: Macrocyclic lactone
mM: Mili molar
μM: Micro molar
MOX: Moxidectin
mRNA: Messenger ribonucleic acid
MRP: Multidrug resistance protein
MW: Molecular weight
NBD: Nucleotide binding domain
PAFOH: Paraformaldehyde
PBS: Phosphate buffered saline
PCR: Polymerase chain reaction
PG: Pablo Godoy
PGP: P-glycoprotein
PMSF: Phenylmethylsulfonyl fluoride
PVDF: Polyvinyl difluoride
qRT-PCR: Quantitative real-time PCR
Rho123: Rhodamine 123
RKP: Roger K. Prichard
RNA: Ribonucleic acid
RNB: Robin N. Beech
RT-PCR: Reverse-transcription PCR
SOP: Standard operating procedure
TBS: Tris buffered saline
TMD: Transmembrane domain
Tris–HCl: Tris(hydromexymethyl) aminomethane
U: Units (enzyme)
VSP: Valspodar
v/v: Volumen/volumen
v/w: Volumen/weight
WB: Western-blot
