# Supplementary material for: Characterisation of P-glycoprotein-9.1 in Haemonchus contortus
Source: Parasit Vectors. 2016 Jan 28;9:52. doi: 10.1186/s13071-016-1317-8 (PMC4730751; doi:10.1186/s13071-016-1317-8)
Supplement: Additional file 1: — Multiple sequence alignment of Hco -PGP-9.1 (GenBank accession number JX430397), Hco -PGP-9.2 (GenBank accession number: JX430398) and Hco -PGP-9.3 (GenBank accession number: JX430395). (DOC 37 kb) [file 13071_2016_1317_MOESM1_ESM.doc]

Supplementary data 1. Multiple sequence alignment of ***Hco*-PGP-9.1** (Accession number: JX430397), ***Hco*-PGP-9.2** (Accession number:

JX430398) and ***Hco*-PGP-9.3** (Accession number: JX430395).

*Hco*-PGP-9.3 ------------------------------------------------------------ *Hco*-PGP-9.1 MGFLKKNGKVADSKGQDDSQIEGEKKEDAPKASIIQLFRYTTTFDKVLLLIGSFVAIGTG *Hco*-PGP-9.2 ------------------------------------------------------------

*Hco*-PGP-9.3 ------------------------------------------------------------ *Hco*-PGP-9.1 IGLPMMSIIMGNISQNFMNINGNTTTINQFEHDVIQNCLKYVYLGCGIFTAATIQAICFL *Hco*-PGP-9.2 ------------------------------------------------------------

*Hco*-PGP-9.3 ------------------------------------------------------------ *Hco*-PGP-9.1 TVCENLVNQLRRQFFKSILRQDITWFDKNNSGTLATKLFDNLERVKEGTGDKLGLMIQFV *Hco*-PGP-9.2 ------------------------------------------------------------

*Hco*-PGP-9.3 ------------------------------------------------------------ *Hco*-PGP-9.1 AQFFGGFIVAFTYDWKLTLIMMSLAPFMIICGAFIAKLMATAATREAKKYAVAGGIAEEV *Hco*-PGP-9.2 ------------------------------------------------------------

*Hco*-PGP-9.3 ------------------------------------------------------------ *Hco*-PGP-9.1 LTSIRTVIAFNGQPYECERYQKALEDGKSTGIKKSFYIGVGLGITFLIMFSSYCLAFWVG *Hco*-PGP-9.2 ------------------------------------------------------------

*Hco*-PGP-9.3 ------------------------------------------------------------ *Hco*-PGP-9.1 TDFVFKGQMNGGTVMTVFFSVMMGSMALGQAGPQFAVLGTAMGAAGSLYQIIDREPEIDS *Hco*-PGP-9.2 ------------------------------------------------------------

*Hco*-PGP-9.3 ------------------------------------------------------------ *Hco*-PGP-9.1 YSTDGVKPSNLKGKVTVSNLKFTYPTRPDVPILKGVSFEANPGETIALVGSSGCGKSTII *Hco*-PGP-9.2 ------------------------------------------------------------

*Hco*-PGP-9.3 ------------------------------------------------------------ *Hco*-PGP-9.1 QLLLRYYNPEDGKITIDGVEIDKINIEFLRNYVGVVSQEPMLFNTTIEQNIRYGRENVTD *Hco*-PGP-9.2 ------------------------------------------------------------

*Hco*-PGP-9.3 ------------------------------------------------------------ *Hco*-PGP-9.1 AEITAALRKANAYNFVQSFPDGIYTNVGDRGTQMSGGQKQRIAIARALVRDPKILLLDEA *Hco*-PGP-9.2 ------------------------------------------------------------

*Hco*-PGP-9.3 ------------------------------------------------------------ *Hco*-PGP-9.1 TSALDAESEHIVQQALENASKGRTTIVVAHRLSTIRNADKIVAMKNGEVMEVGTHDELIA *Hco*-PGP-9.2 ------------------------------------------------------------

*Hco*-PGP-9.3 ------------------------------------------------------------ *Hco*-PGP-9.1 RKGLYHELVNAQVFADVDDKSGEPGDRRRTMSSSRSRSPSLASPEYKRLKSQMSTEIAAG *Hco*-PGP-9.2 ------------------------------------------------------------

*Hco*-PGP-9.3 ------------------------------------------------------------ *Hco*-PGP-9.1 GGAQNDPVKAEKDLERLKKELEEEGAAKANLFKILGYARPEWPFIALAVTSSIVQGCVFP *Hco*-PGP-9.2 ------------------------------------------------------------

*Hco*-PGP-9.3 ------------------------------------------------------------

*Hco*-PGP-9.1 AFSLFFSQIIDVFSKQPGDPTLKSDGHFWALMFLVLGGTQAMTMLIQCFFFGLSAERLTM

*Hco*-PGP-9.2 ------------------------------------------------------------

*Hco*-PGP-9.3 ------------------------------------------------------------

*Hco*-PGP-9.1 RLRSKIFQNVMRMDATYFDMPRHSAGKITTRLATDAPNVKSALDYRFGSVFSSVVSICCG

*Hco*-PGP-9.2 --------------------------------------------YRFGSVFSSVVSICCG

*Hco*-PGP-9.3 ------------------------------------------------------------

*Hco*-PGP-9.1 IGIAFYFGWQMALLTIAIFPLAAVGQAIQMRFMSGRATADAKEMENSGKIAMEAIENIRT

*Hco*-PGP-9.2 IGIAFYFGWQMALLTIAIFPLAAVGQAIQMRFMSGRATADAKEMENSGKIAMEAIENIRT

*Hco*-PGP-9.3 ------------------------------------------------------------

*Hco*-PGP-9.1 VQALTLERRLHAQFCHHLDGPHKTSRRKALIQGVSYGFASSIFYFLYASCFRFGLWLIVN

*Hco*-PGP-9.2 VQALTLERRLHAQFCHHLDGPHKTSRRKALIQGVSYGFASSIFYFLYASCFRFGLWLIVN

*Hco*-PGP-9.3 ------------------------------------------------------------

*Hco*-PGP-9.1 GTIHSMNVLRVLFAISFTAGSMGFASSYFPEYIKATFAAGIIFHMLEEEPRIDGMTNNGK

*Hco*-PGP-9.2 GTIHSMNVLRVLFAISFTAGSMGFASSYFPEYIKATFAAGIIFHMLEEEPRIDGMTNNGK

*Hco*-PGP-9.3 ------------------------------------------------------------

*Hco*-PGP-9.1 KPKITGAVKLNKVYFKYPERPDVPILQGLDINVKPGETLALVGPSGCGKSTVISLLERLY

*Hco*-PGP-9.2 KPKITGAVRLNKVYFKYPERPDVPILQGLDINVKPGETLALVGPSGCGKSTVISLLERLY

*Hco*-PGP-9.3 -------EVDGNDLREMNPTHLRAHIALVSQEPILFDRSIRDNILYGLPPGSVSEAEVHE

*Hco*-PGP-9.1 DALDGSVEIDGNDLREVNPTHLRAHIALVSQEPILFDRSIRDNILYGLPPGSVSDAAVHE

*Hco*-PGP-9.2 DALDGSVEIDGNDLREVNPTHLRAHIALVSQEPILFDRSIRDNILYGLPPGSVSDAAVHE

*:*******:*************************************:* ***

*Hco*-PGP-9.3 VAQRANIHKFVMELPEGYNTRAGEKGVQLSGGQKQRIAIARALIRNPKILLLDEATSALD

*Hco*-PGP-9.1 VAQRANIHKFIMDLPDGYNTRAGEKGTQLSGGQKQRIAIARALIRNPKILLLDEATSALD

*Hco*-PGP-9.2 VAQRANIHKFIMDLPDGYNTRAGEKGTQLSGGQKQRIAIARALIRNPKVLLLDEATSALD

**********:*:**:**********.*********************:***********

*Hco*-PGP-9.3 TESEKVVQEALDKASEGRTCIVVAHRLSTVVNANCIMVVQGGKIVEKGTHNELMQAKGVY

*Hco*-PGP-9.1 TESEKVVQEALDKASEGRTCIVVAHRLSTVVNANCIMVVKGGKVVEKGTHSELMQAKGAY

*Hco*-PGP-9.2 TESEKVVQEALDKASEGRTCIVVAHRLSTVVNANCIMVVKGGKVVEKGTHSELMQAKGAY

***************************************:***:******.*******.*

*Hco*-PGP-9.3 WELTQKQTTAKE

*Hco*-PGP-9.1 WALTQKQTLAKG

*Hco*-PGP-9.2 WALTQKQTLAKE

* ****** **

*Hco*-PGP-9.1 epitopes selected by 21^st^ Century Biotech. Inc. (Malborough, MA, USA) to generate antibody. Epitope (1) corresponds to aa 648-674, and epitope (2) corresponds to aa 1042-1060. Both are intra-cytoplasmic locations on *Hco*-PGP-9.1. Protein translation from the cloned DNA was carried out with Expasy translate tool (http://web.expasy.org/translate/). Alignment with *Hco*-PGP-9.2 and *Hco*-PGP-9.3 amino acid (partial) sequences, executed using MUSCLE (3.8) (http://www.ebi.ac.uk/Tools/msa/muscle/)
